# Mass sterilization of a common palm species by elephants in Kruger National Park, South Africa

**DOI:** 10.1038/s41598-020-68679-8

**Published:** 2020-07-16

**Authors:** Jeremy J. Midgley, Bernard W. T. Coetzee, Donovan Tye, Laurence M. Kruger

**Affiliations:** 1 Department of Biological Sciences, University of Cape Town, P Bag Rondebosch, Cape Town, 7701 South Africa; 2 Organization for Tropical Studies, P. Bag, Skukuza, 1350 South Africa; 3 Global Change Institute, University of the Witwatersrand, Wits, Johannesburg, 2050 South Africa

**Keywords:** Ecology, Plant sciences

## Abstract

Chronic herbivory by elephants rarely eliminates any species of woody savanna plants because these plants are typically vigorous basal resprouters after damage by fire or herbivory. In some instances, resprouting after elephant herbivory even increases stem numbers per unit area compared to protected areas. It is thus difficult to know whether an area has been severely degraded by elephant herbivory or not because although trees may be severely reduced in size, they will still be present and may even be relatively dense. By using an elephant exclosure in the Kruger National Park, South Africa, we demonstrate that this resprouting ability masks the fact that entire populations of a widespread African palm, *Hyphaene petersiana*, are prevented from reaching sexual maturity by chronic elephant herbivory. Besides sterilizing these palms and thus preventing their evolution and seed dispersal, the absence of the palm fruits, flowers and tall stems has other negative biodiversity impacts on their associated fauna. We suggest that to determine sustainable elephant impacts on savanna plants, conservation managers also use the reproductive condition of savanna plants rather than their presence, height or stem density.

## Introduction

The conservation of African savannas both with and without elephants is controversial; the species is in drastic decline in many places, but in other areas, such as the Kruger National Park (KNP) South Africa, they are increasing^1^. Given this general decline in elephants good evidence needs to be presented that populations need to be reduced to prevent irreversible vegetation change. In the decades prior to the 1970s rapid increases in African elephant populations occurred in nature reserves in several countries and this led to “the elephant problem”; the widespread conversion of woodlands to grasslands^2,3^. Whether the loss of woodlands was permanent or even a problem and whether elephant numbers should be reduced were controversial. For example, Caughley^3^ argued that as elephant populations increased, they would cause woody plant abundance to decline. Because elephants prefer browse and need shade^2^, this decline would then cause elephant populations to crash and subsequently woodlands would recover but that this would occur at the scale of centuries. This hypothesis is based on the observation that woodland species such as mopane (*Colophospermum mopane*) studied by Caughley^3^ are locally highly persistent; they could resprout and recruit in situ after elephant damage declined. Also, it implies either that elephants can disperse widely or that elephant populations can crash. These are unlikely events in present parks. Also, some species such as baobabs (*Adansonia digitata*) and various African *Acacia* species are less tolerant of chronic elephant damage^2,3^ including at KNP^4^. They would only recover, if at all, with substantial periods of very low elephant impacts and high levels of seed dispersal. Some savanna species require elephants for seed dispersal^5–9^ and these species would be disadvantaged by an absence of elephants. Thus, the determination of appropriate elephant numbers in savanna reserves is complex.

For example, despite the having had a long history of scientific research and management of elephants and their impacts^10^, determining the carrying capacity of the rapidly expanding KNP population is still controversial^10–14^. Herbivory by elephants strongly impacts KNP vegetation, largely by reducing the height of plants^10,11^ but only rarely by causing local plant extinction (only the 2–4 m tall succulent *Aloe marlothii*)^10^. Because most savanna plants, from grasses to trees, can resprout from their base, stems or roots, they are highly tolerant of disturbance by elephants, by fire and by both. This persistence^15^ and thus relative lack of plant extinction and thus compositional change due to elephant damage, has led conservators to debate what constitutes the desired state of savanna vegetation, such as of vegetation height and structural heterogeneity^10,12,13^ as well as the biodiversity consequences of these, such as on other fauna^16^. Here we use the tall (18 m) common savanna woody palm species *Hyphaene petersiana* to re-consider savanna conservation in KNP. It is highly appropriate for this as it is both browsed and dispersed by elephants^7–9^. *H. petersiana* is considered to be resistant to chronic elephant herbivory in KNP because its stem density was greater outside an elephant exclosure than within the exclosure^12^. This species responds to browsing and stem damage by elephants, with the prolific production of new stems by root resprouting and therefore is seen as highly resilient. Here we consider the impact of loss of stem size on its capacity to produce fruit and flowers.

Given that most plants need to reach a minimum size to be reproductive and that this minimum size is proportional to the maximum size of the species^17,18^, we compared the reproductive status of individuals of a tall (18 m) palm species that were exposed to, or protected from, elephant herbivory in KNP. Direct elephant impacts on the reproductive status of resprouting plant populations have not previously been studied, although Caughley^3^ noted that seeding of mopane (*Colophospermum mopane*) in Zambia was more strongly determined by stem height than girth. Thus, the probability that a mopane stem can produce seeds if kept at a height of 2 m by elephant damage, despite a girth of 0.5 m, 1.0 m or 1.5 m, was only 0.5%, 1% and 2% respectively.

## Results and discussion

**Figure 1:**
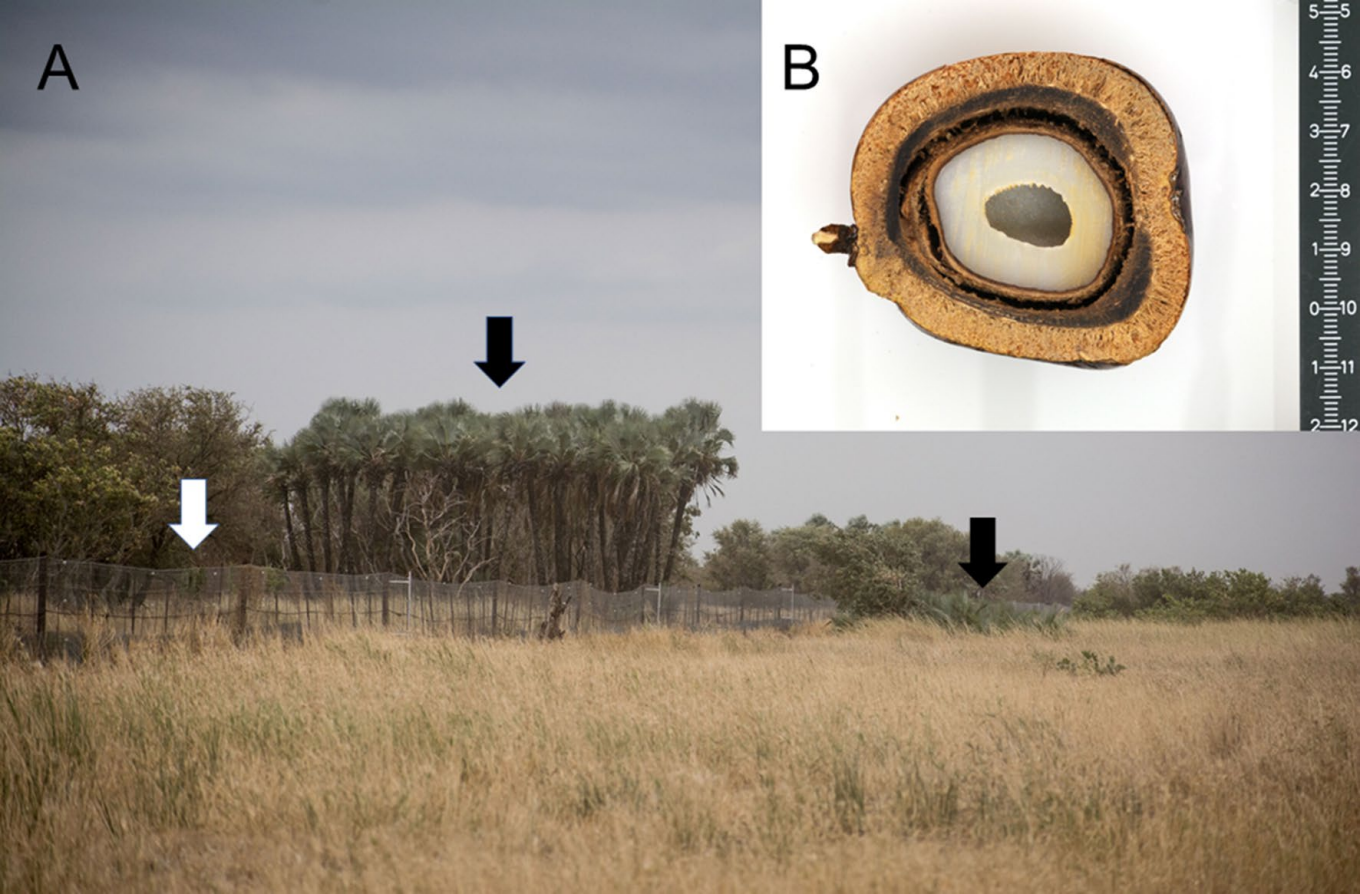
Arrows indicate short *H. petersiana* palms outside the 2 m tall electric fence compared to tall palms within the exclosure (**A**). The large fruits of *H. petersiana* (**B**).

**Figure 2:**
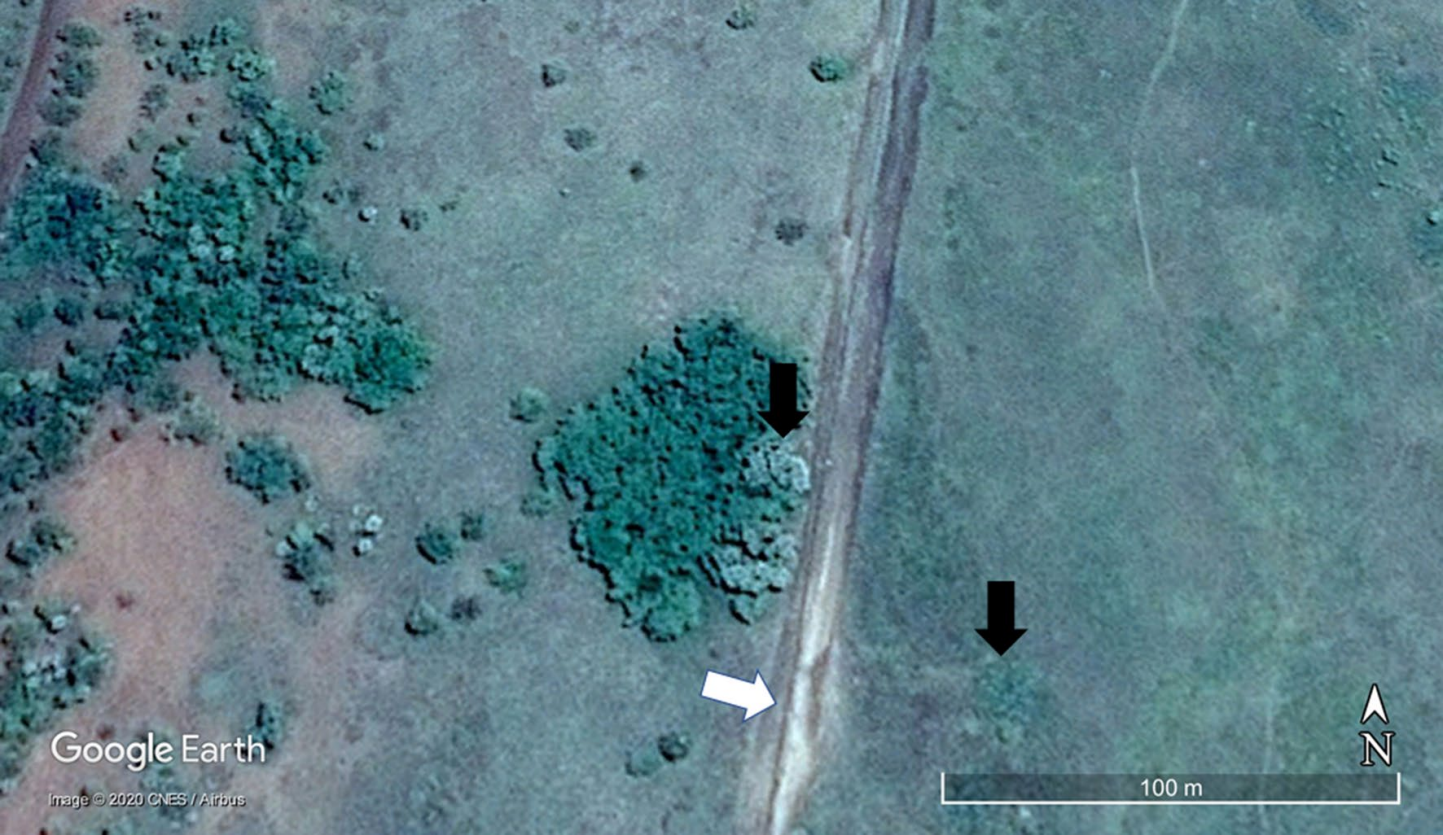
Arrows indicate that on Google earth image (Image 2013 CNES/Airbus) of where Fig. 1 was taken, tall palms are clearly visible within the exclosure (grey-green canopies) but are short outside the fence.

**Figure 3:**
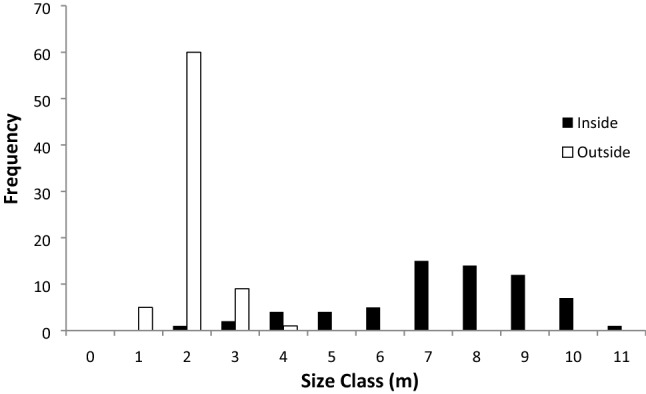
Size-class distribution of *H. petersiana* inside and outside the elephant exclosure.

Elephant herbivory in KNP presently prevents a widespread woody palm *Hyphaene petersiana* from reaching reproductive size. Out of 65 individual palms sampled inside the Nwaxitshumbe elephant exclosure, 60 (32 females, 28 males) were mature (92%). The mean maximum height of individuals within the enclosure was 7.0 m (range 1.5–11 m). This palm reaches maturity between 4–5.3 m in height as evidenced by the mean height of the tallest immature stems per individual as 5.3 m and the mean height of the shortest mature stems as 4 m (n = 20). Outside the exclosure the mean height of the 75 surveyed individuals was only 1.6 m (max 3.2 m, only 30% > 2 m). Not one of these were reproductive, with most being several (2.5+) m short of being reproductive (Figs. 1, 2, 3). Signs of elephant herbivory of the palm outside the exclosure were widespread, as has been found elsewhere in Africa^19^. We found no seedlings inside or outside the exclosure (Fig. 3). Outside the exclosure this is due to a widespread lack of reproduction. The absence of elephants and their role in dispersal and germination^7–9^ explain the lack of recruitment inside the exclosure, despite the production of many thousands of fruits annually over several decades.

This simple result of mass sterilization by elephants is important for biodiversity conservation for at least three reasons. Firstly, and critically, sterile plants cannot evolve new adaptations, such as to the looming threat of global change, nor can they disperse seeds to move with moving climate zones. Secondly, without seedling recruitment populations will eventually go extinct, although in the case of this highly persistent resprouting palm, this is only likely with sudden or significant environmental change because this species can live for about a century^20^. Thirdly, because sterile plants do not produce flowers, fruits and large stems this too has biodiversity implications. We observed ad hoc that the outer layer of the fruits of this palm (Fig. 1) is eaten by vervet monkeys (*Chlorocebus pygerythrus*), porcupines (*Hystrix africaeaustralis)* and squirrels (*Paraxerus cepapi*). Elephants also consume *Hyphaene* fruits^7–9^. We observed the palm flowers to be heavily visited by pompilid wasps, that African palm swifts (*Cypsiurus parvus*) were only nesting in tall palms inside the exclosure and that woodpeckers used the tall soft stems for nest sites. Sterilization therefore has diverse biodiversity consequences. These negative impacts are based on data from one location and for only one plant species, but these impacts are likely geographically widespread and to occur on other common woody KNP species. As minimum size to maturity in plant species is well known to scale with their maximum height^17,18^ and therefore broken, but potentially tall trees are likely sterile, as was the case for *H. petersiana*. For example, the geographically widely-distributed important savanna tree *Colophospermum mopane* (“mopane”) can reach 10–25 m tall but is most often a short (< 2 m), broken tree described as being “planed”, “hedged”, “dwarfed” or “bonsaied” by elephants in KNP and elsewhere^3,21,22^. An extensive (> 60 km transect) google earth survey of *H. petersiana* showed an almost total absence of mature individuals outside of elephant exclosures and a survey of a population of 40 individuals of the congener *H. coriacea,* indicated that 75% of individuals were sterile.

Since there are only a few antelope (approximately 8 ha per animal during the period 2000–2017 according to SanParks records) within the exclosure, grass biomass is much higher inside than outside. The exclosure is actively burned to maintain the grazing for these rare antelope and although many of the palms inside the exclosure had been burned recently, their canopies had escaped damage because they are several metres above the high grass-biomass fueled fire zone. Many fruits on the ground below mature individuals were damaged by the fire. Outside the exclosure elephant herbivory keeps plants short and therefore when fires take place, fire damages fronds and this exacerbates the lack of plants becoming tall enough to become reproductive. The achievement of reproductive size inside the exclosure is due to the absence of elephants rather than an absence of fire.

The impact of elephant herbivory on reducing the size of this palm outside compared to inside the Nwaxitshumbe exclosure was previously noted by Levick and Rogers^12^. However, they interpreted elephant herbivory as having a positive impact on this palm, because of a greater relative stem density outside the exclosure. Also, they suggested that tall vegetation in the exclosure “would be less permeable to vectors such as wind and water”^12^. They missed the dramatic and negative impact on reproductive status despite *H. petersiana* fruits being conspicuously large (up to 10 cm in length) and individual fruit-loads often exceed 100 fruits (Fig. 1). We suggest this was missed because assessing reproductive condition is not a routine conservation assessment of the impacts of herbivory. The debatable positive impacts of elephant herbivory on this palm suggested by Levick and Rogers^12^ should be weighed up against more definitely negative impacts on the reproductive status of plants and the additional negative impacts this has, for example on frugivores and pollinators. We suggest that managers consider the conservation impacts of elephants, both positive and negative, on the sexual reproduction of resprouting plants. Although fruiting is less obvious for most plant species than for *H. petersiana*, given its large fruits, it would nevertheless be relatively easy to assess the minimum size a species needs to be, to be sexually reproductive. Species with tall maximum heights may be a priority. Also, if the present high level of elephant herbivory in KNP is reduced, fruiting by well-established resprouts of this palm could occur within two decades, because they are capable of rapid growth^20^. However, there is no plan^14^ to directly control the presently steadily increasing population^1^, although there are plans to reduce access to artificial waterpoints^14^ Finally, we emphasize the general conservation problem that resprouting plant species such as *H. petersiana* present^15^. Although they are able to increase stem density despite chronic elephant herbivory or persist in situ in the absence of elephants, their loss of reproduction or loss of their dispersal mutualists, means that they are nevertheless presently “the living dead”^23^ in KNP.

## Methods

We compared plant height and reproductive status (flowering and/or fruiting) of the widespread African palm *Hyphaene petersiana* (lala palm, vegetable ivory palm) inside and outside the Nwaxitshumbe exclosure (22° 46ʹ 44.48″ S, 31° 15ʹ 32.24″ E; Fig. 1) in the northern KNP. The 302 ha exclosure was built to protect rare antelope (*Hippotragus equinus* and *Alcelaphus buselaphus*) and it thus excludes all mammals larger than a hare, especially elephant. The exclosure where the palm occurs was erected in 1986^12^. Evidence that the palms within the exclosure were released from elephant herbivory after it was erected in 1986 is that they were shorter after 15 years of protection^12^ than in our survey after 33 years (Figs. 2, 3) and that the palm requires elephants for seedling recruitment^7–9^. Elephant numbers in KNP have increased since the 1960s and especially in the 2000s after culling ended in 1995^14^ and they continue to rise^1^.

To discern whether an individual was mature or not, we sampled this multi-stemmed dioecious palm during the flowering season*. H. petersiana* is a vigorous resprouter and produces a clump of many basal and root sprouts (ramets) which together constitute a genetical individual (genet). Without genetical tools, it is difficult to know where a genet begins or ends. We used two indices of the presence of a new genet; stems more than 20 m from the nearest *H. petersiana* stem or stems from a different sex. Based on our survey we suggest 20 m is a conservative estimate as we often had different sexes within 5–10 m of each other and our measured sex ratio (1.14 f:1 m) is close to the expected 1:1. For each individual clump we estimated the height of the tallest stem of the clump and assessed whether it was sexually mature and if so, the sex. To determine the approximate minimum size at maturity, for the same clump we also noted the dimensions of the tallest non-reproductive stem and the dimensions of the shortest reproductive stem. Two observers estimated height based on a standard 2 m length. For all individuals we sampled height to the apex of the tallest frond. We sampled all individuals (genets) within the enclosure and 25 individuals in each of 3 transects outside the enclosure. We searched for seedlings defined as plants < 1 m tall and with characteristic juvenile foliage (all the data available as Table S1). Levick and Rogers^12^ sampled all stems in five plots (each 2.5 m × 20 m) within and outside the exclosure and hence their data includes mainly ramets.

To demonstrate that this sterilization is widely geographically distributed we also sampled *H. petersiana* remotely. It is widespread in KNP and southern Africa along rivers and shallow floodplains (“vleis”)^24^. Tall individuals are observable on google earth (Fig. 2) but small resprouting individuals are not. We searched for tall individuals in a 60 km strip along the road from to Shingwedzi Rest Camp (23ʹ 06″ 51S, 31ʹ 28″ 06E) to the Nwaxitshumbe exclosure using both road surveys and google Earth surveys. Despite this transect passing large populations in many vleis and rivers, we only found three tall individuals (in a steep riverbank), out of an estimated several million individuals. Using the same method, we also sampled a population of 40 individuals of the congener *H. coriacea* at Letaba (24ʹ 18″ 32S, 31ʹ 44″ 49E) in KNP.

## Supplementary information

Supplementary Table S1.
