# Supplementary material for: Mass sterilization of a common palm species by elephants in Kruger National Park, South Africa
Source: Sci Rep. 2020 Jul 16;10:11719. doi: 10.1038/s41598-020-68679-8 (PMC7366642; doi:10.1038/s41598-020-68679-8)
Supplement: Supplementary file 1 — Supplementary Table S1. [file 41598_2020_68679_MOESM1_ESM.pdf]

Mass sterilization of a common palm species by elephants in Kruger National Park, South Africa.

Jeremy J. Midgley[1*], Bernard W.T. Coetzee[2,3], Donovan Tye[2] & Laurence M. Kruger[1,2]

1. Department of Biological Sciences, University of Cape Town, P bag Rondebosch, 7701, South Africa

2. Organization for Tropical Studies, P. Bag, Skukuza, 1350, South Africa

3. Global Change Institute, University of the Witwatersrand, Johannesburg, Wits 2050, South Africa

* Jeremy.Midgley@uct.ac.za

Table S1. Raw data used for this study of *Hyphaene petersiana* collected inside (Y) and outside (N) the Nwaxitshumbe exclosure in the Kruger National Park, South Africa. Height is metres.

Exclosure	Clump	Central Stem?	Sex	Fruit/flowers?	Mature Tallest Height	Shortest Mature Height	Tallest Immature Height
Y	1	N	N/A	N	N/A	N/A	1.5
Y	2	Y	F	Y	10	N/A	N/A
Y	3	Y	F	Y	5	3.5	N/A
Y	4	Y	F	Y	8	4	N/A
Y	5	Y	M	Y	7.5	5	6
Y	6	Y	F	Y	9.5	9	9
Y	7	Y	M	Y	9.5	9	5.5
Y	8	Y	M	Y	11	9.5	N/A
Y	9	Y	M	Y	7	4	5
Y	10	Y	F	Y	5	6.25	7.25
Y	11	Y	M	Y	4.2	N/A	N/A
Y	12	Y	F	Y	5	N/A	5
Y	13	Y	N/A	N	N/A	N/A	3.5
Y	14	Y	M	Y	8.5	N/A	3
Y	15	Y	F	Y	7.25	3	2.5
Y	16	Y	M	Y	6	5.5	2.2
Y	17	Y	M	Y	7.5	5	7
Y	18	Y	N/A	N	N/A	N/A	4
Y	19	Y	M	Y	8.5	3.5	2
Y	20	Y	F	Y	10	7.5	7
Y	21	Y	M	Y	8.5	7	1
Y	22	Y	F	Y	5.5	3	1
Y	23	Y	F	Y	6.5	6	2.5
Y	24	Y	M	Y	7.5	N/A	2
Y	25	Y	F	Y	8.5	6.5	5
Y	26	Y	M	Y	6.5	N/A	4.3
Y	27	Y	F	Y	8	N/A	N/A

Y	28	Y	M	Y	8.5	N/A	N/A
Y	29	Y	F	Y	7	N/A	N/A
Y	30	Y	M	Y	6.5	N/A	4.7
Y	31	Y	M	Y	6.5	4.5	N/A
Y	32	Y	F	Y	6.3	N/A	2.5
Y	33	Y	M	Y	7	N/A	N/A
Y	34	Y	F	Y	6.2	N/A	5
Y	35	Y	F	Y	5	N/A	N/A
Y	36	Y	M	Y	8	5	N/A
Y	37	Y	F	Y	8	N/A	N/A
Y	38	Y	F	Y	7.5	4.1	N/A
Y	39	Y	M	Y	7	5.8	N/A
Y	40	Y	F	Y	8.4	6	N/A
Y	41	Y	M	Y	9.8	3.7	N/A
Y	42	Y	F	Y	10	3.9	N/A
Y	43	Y	M	Y	9	N/A	N/A
Y	44	Y	F	Y	6.2	6	N/A
Y	45	Y	F	Y	N/A	4	N/A
Y	46	Y	M	Y	6.6	N/A	5
Y	47	Y	F	Y	8.5	N/A	3.8
Y	48	Y	M	Y	8.2	N/A	N/A
Y	49	Y	F	Y	6	5.5	N/A
Y	50	Y	F	Y	9	6	3.5
Y	51	Y	F	Y	8	8	N/A
Y	52	Y	N/A	N	N/A	N/A	2.5
Y	53	Y	M	Y	7	4	N/A
Y	54	Y	M	Y	10	8	N/A
Y	55	Y	M	Y	8	7	4
Y	56	Y	F	Y	7	N/A	N/A
Y	57	Y	M	Y	6	4	4
Y	58	Y	F	Y	6	5.5	4
Y	59	Y	F	Y	8.5	6	5
Y	60	Y	F	Y	4	4	N/A
Y	61	Y	M	Y	8	3.5	N/A
Y	62	N	N/A	N	N/A	N/A	2.2
Y	63	Y	F	Y	8	6	5
Y	64	Y	F	Y	9	4	5
Y	65	Y	M	Y	7	4	N/A
N	1	N	N/A	N	N/A	N/A	1.75
N	2	N	N/A	N	N/A	N/A	1.75
N	3	N	N/A	N	N/A	N/A	1.75
N	4	N	N/A	N	N/A	N/A	1.5
N	5	N	N/A	N	N/A	N/A	1.5
N	6	N	N/A	N	N/A	N/A	1.5
N	7	N	N/A	N	N/A	N/A	1.2
N	8	N	N/A	N	N/A	N/A	1.4
N	9	N	N/A	N	N/A	N/A	1.7
N	10	N	N/A	N	N/A	N/A	1.4

N	11	N	N/A	N	N/A	N/A	1.7
N	12	N	N/A	N	N/A	N/A	1
N	13	N	N/A	N	N/A	N/A	1.3
N	14	N	N/A	N	N/A	N/A	1.5
N	15	N	N/A	N	N/A	N/A	1.7
N	16	N	N/A	N	N/A	N/A	1.7
N	17	N	N/A	N	N/A	N/A	1.1
N	18	N	N/A	N	N/A	N/A	1.1
N	19	N	N/A	N	N/A	N/A	1.2
N	20	N	N/A	N	N/A	N/A	1.2
N	21	N	N/A	N	N/A	N/A	1.2
N	22	N	N/A	N	N/A	N/A	1.9
N	23	N	N/A	N	N/A	N/A	2
N	24	N	N/A	N	N/A	N/A	1.2
N	25	N	N/A	N	N/A	N/A	1.75
N	26	N	N/A	N	N/A	N/A	1.5
N	27	N	N/A	N	N/A	N/A	1.75
N	28	N	N/A	N	N/A	N/A	2
N	29	N	N/A	N	N/A	N/A	2
N	30	N	N/A	N	N/A	N/A	1.9
N	31	N	N/A	N	N/A	N/A	2
N	32	N	N/A	N	N/A	N/A	2.3
N	33	N	N/A	N	N/A	N/A	2
N	34	N	N/A	N	N/A	N/A	2.25
N	35	N	N/A	N	N/A	N/A	2.1
N	36	N	N/A	N	N/A	N/A	2.5
N	37	N	N/A	N	N/A	N/A	2
N	38	N	N/A	N	N/A	N/A	1.5
N	39	N	N/A	N	N/A	N/A	2.3
N	40	N	N/A	N	N/A	N/A	1.9
N	41	N	N/A	N	N/A	N/A	3.2
N	42	N	N/A	N	N/A	N/A	2
N	43	N	N/A	N	N/A	N/A	2
N	44	N	N/A	N	N/A	N/A	1.8
N	45	N	N/A	N	N/A	N/A	1.9
N	46	N	N/A	N	N/A	N/A	2.25
N	47	N	N/A	N	N/A	N/A	1.9
N	48	N	N/A	N	N/A	N/A	1.85
N	49	N	N/A	N	N/A	N/A	2.2
N	50	N	N/A	N	N/A	N/A	1.5
N	51	N	N/A	N	N/A	N/A	2
N	52	N	N/A	N	N/A	N/A	1.5
N	53	N	N/A	N	N/A	N/A	1.25
N	54	N	N/A	N	N/A	N/A	1.6
N	55	N	N/A	N	N/A	N/A	1.5
N	56	N	N/A	N	N/A	N/A	1.2
N	57	N	N/A	N	N/A	N/A	1.5
N	58	N	N/A	N	N/A	N/A	1.8

N	59	N	N/A	N	N/A	N/A	2
N	60	N	N/A	N	N/A	N/A	2.2
N	61	N	N/A	N	N/A	N/A	1.2
N	62	N	N/A	N	N/A	N/A	1.75
N	63	N	N/A	N	N/A	N/A	1
N	64	N	N/A	N	N/A	N/A	1.1
N	65	N	N/A	N	N/A	N/A	2.3
N	66	N	N/A	N	N/A	N/A	1
N	67	N	N/A	N	N/A	N/A	1
N	68	N	N/A	N	N/A	N/A	1.9
N	69	N	N/A	N	N/A	N/A	1
N	70	N	N/A	N	N/A	N/A	1.1
N	71	N	N/A	N	N/A	N/A	2
N	72	N	N/A	N	N/A	N/A	2
N	73	N	N/A	N	N/A	N/A	1.1
N	74	N	N/A	N	N/A	N/A	1.7
N	75	N	N/A	N	N/A	N/A	1.5
